# Welcome to Volume 9 of *CNS Oncology*

**DOI:** 10.2217/cns-2019-0023

**Published:** 2020-03-02

**Authors:** Jennifer Straiton

**Affiliations:** 1 Future Science Group, Unitec House, 2 Albert Place, London, N3 1QB, UK

To all of our readers, Happy New Year and welcome to the first issue of Volume 9 of *CNS Oncology*. 2020 promises to be an exciting year and we look forward to growing the reach and audience of the journal as we enter the new decade. However, before we start this new period, I would like to look back over the past year and our highlights from 2019.

## Content highlights

Our most-read article of the year comes from Ugonma Chukwueke and Patrick Wen, both of the Dana-Farber Cancer Institute (MA, USA), and reviews the recommendations of the Response Assessment in Neuro-Oncology (RANO) working groups [1]. Distinct RANO groups have been set up for different tumor and treatment types, each group aiming to develop relevant and reliable criteria that can be implemented by clinicians. This review discusses how the recommendations given by the RANO groups may be applied to the care of patients with different brain tumor types and suggests what is still needed for the guidelines to fully reflect the complex nature of each tumor. For anyone interested in neuro-oncology and CNS oncology management, this is a key read. From Issue 1 of this volume, the article has been cited twice and is already the most-read article ever published in *CNS Oncology*.

Next up, we have a preliminary communication written by David E Piccioni *et al.* (University of California San Diego, CA, USA), describing their study evaluating whether circulating tumor DNA could facilitate genomic interrogation in patients with primary brain tumors [2]. Contrary to previous studies in this area, the group found that 50% of patients included in the study had detectable circulating tumor DNA, making it a clinically viable option for analysis when identifying genomics-based therapy options.

Another 2019 article that has hit our most-read list is an editorial that evaluates the benefits of whole-brain radiation therapy versus stereotactic radiosurgery for the treatment of brain metastases [3]. Written by a group from the City of Hope National Medical Center (CA, USA), the article discusses the shift in clinical practice from using whole-brain radiation therapy to stereotactic radiosurgery and looks to the future of radiation therapy for brain metastases.

Finally, we have a set of three articles each discussing the Nativis Voyager^®^, an investigational therapeutic medical device that is being developed for the treatment of both pediatric and adult glioblastoma. With feasibility studies in both American [4] and Australian [5] populations, the researchers found no adverse events that could lead to the discontinuation of the device, and it demonstrated a benign safety profile. However, as stated in an editorial on the device by Victor Levin (MD Anderson, TX, USA), further studies are needed to determine the full impact of the therapy on overall survival of patients with glioblastoma [6].

## Editorial board

To our editorial board, we thank you for your continued input, be it in an ambassadorial, advisory or authorship role. Led by our senior editors Alba Brandes (Azienda USL, Bologna, Italy) and Henry Friedman (Duke University Medical Center, NC, USA), our international board provides valuable assistance and advice that facilitate the publication process; we look forward to working together in the coming year.

On this note, if you are interested in joining our editorial board or wish to provide feedback or suggestions for the journal, please do not hesitate to get in touch; we value any and all input that can contribute to the growth and development of the journal.

## Article outreach

To help further spread the work we publish, we continue to utilize the power of social media and share new work through platforms such as Twitter and LinkedIn to ensure articles can be shared with the largest possible audience; if you do not already, we welcome you to follow us on Twitter (@fsg_cns) or join our oncology group on LinkedIn, Future Science Group Oncology. In using social media, it is our aim to reach all relevant stakeholders in the field of CNS oncology – including researchers, clinicians, charities, patients, academics/educators and patient advocates, just to name a few.

Our partnerships with the sites Oncology Central [7] and Neuro Central [8] continue, giving authors the opportunity to put their work on the websites and be seen by its wide readership base. Registration to both sites is free and allows you to keep up to date with the latest developments in cancer or neurology via unparalleled free access to the latest news, opinion, peer-reviewed journal articles, multimedia and exclusive content. A great example of this partnership can be seen in our ‘peek behind the paper’ interview with Charles Cobb, senior author on one of our Voyager papers. In the interview, hosted on Neuro Central, Cobb gives insight into the inspiration behind the Voyager device and discusses challenges faced in developing it [9].

I am also excited to announce our partnership with the *Video Journal of Biomedicine* [10]. Now, our authors are invited to work with our expert video team to produce a short interview-style video, discussing their research. Our first video interview, featuring author Rimas Lukas (Northwestern University, IL, USA), will be published online soon and will see Lukas discussing his recent article in Issue 3 titled, ‘Pleomorphic xanthoastrocytoma: a brief review’ [11]. Follow our Twitter, or the Video Journal (@VJofBiomedicine), for updates when this is published.

## Conclusion

Our readers remain central to the success of our journal and we welcome any feedback. Please do not hesitate to contact us with any suggestions for what you would like to see featured or any article proposals of your own. We welcome a wide range of unsolicited article proposals so please do get in touch for more information.

I would like to finish by once again thanking all of the authors and reviewers who helped to make Volume 8 possible; we hope to continue to build on this success and look forward to another great year.
